# Tributyltin perturbs femoral cortical architecture and polar moment of inertia in rat

**DOI:** 10.1186/s12891-021-04298-2

**Published:** 2021-05-07

**Authors:** Mingjun Li, Dong Cheng, Hui Li, Wenhuan Yao, Dongmei Guo, Shu’e Wang, Jiliang Si

**Affiliations:** 1 Department of Environmental Health, School of Public Health, Cheeloo College of Medicine, Shandong University, 44 Wenhua Xi Lu, Jinan, 250012 Shandong China; 2 Department of Toxicology, Shandong Center for Disease Control and Prevention, Jinan, China

**Keywords:** Tributyltin, Adipogenesis, Osteogenesis, Bone geometry, Micro-computed tomography, Polar moment of inertia

## Abstract

**Background:**

Tributyltin, a well-known endocrine disruptor, is widely used in agriculture and industry. Previous studies have shown that tributyltin could cause deleterious effects on bone health by impairing the adipo-osteogenic balance in bone marrow.

**Methods:**

To investigate further the effects of tributyltin on bone, weaned male SD rats were treated with tributyltin (0.5, 5 or 50 μg·kg^− 1^) or corn oil by gavage once every 3 days for 60 days in this study. Then, we analyzed the effects of tributyltin on geometry, the polar moment of inertia, mineral content, relative abundances of mRNA from representative genes related to adipogenesis and osteogenesis, serum calcium ion and inorganic phosphate levels.

**Results:**

Micro-computed tomography analysis revealed that treatment with 50 μg·kg^− 1^ tributyltin caused an obvious decrease in femoral cortical cross sectional area, marrow area, periosteal circumference and derived polar moment of inertia in rats. However, other test results showed that exposure to tributyltin resulted in no significant changes in the expression of genes detected, femoral cancellous architecture, ash content, as well as serum calcium ion and inorganic phosphate levels.

**Conclusions:**

Exposure to a low dose of tributyltin from the prepubertal to adult stage produced adverse effects on skeletal architecture and strength.

**Supplementary Information:**

The online version contains supplementary material available at 10.1186/s12891-021-04298-2.

## Background

Osteoporosis is an emerging medical and socioeconomic threat characterized by systemic impairment of bone mass and microarchitecture that increases the propensity for fragility fractures [1], and has become a serious public health problem [2, 3]. Genetic and environmental factors play key roles in the development of osteoporosis [4]. Mounting evidence obtained from studies on animal models and population studies revealed that exposure to endocrine disruptors (EDs) negatively affected bone health [5–8]. Tributyltin chloride (TBT), a notorious ED, is widely used in wood preservatives, disinfection of industrial circulation water, antifouling coatings for ships and slime treatment in paper mills [9]. Human exposure to TBT occurs mainly through consumption of contaminated dietary sources [9]. In one investigation of Mattos et al. on butyltin (BT) contamination in Northern Chilean coast, the calculated consumption of BT might exceed the tolerable daily intake recommended by European Food Safety Authority in the most contaminated sites [10]. Moreover, high levels of BTs have been detected in human liver tissue [11] and blood samples [12]. Previous studies showed that exposure to high dose TBT (over 10 mg·kg^− 1^) during gestation period caused delayed ossification of the fetal skeleton [13, 14]. Our previous study revealed that exposure to a low dose of TBT (50 μg·kg^− 1^) reduced the femoral bone mineral density (BMD) of rats with a downtrend of biomechanical strength [15].

Although measurement of BMD is an indispensable tool to identify individuals at high risk of injury, bone densitometry affords only a two dimensional areal view of the three dimensional mineralized mass of the skeleton [4]. Micro-computed tomography (μCT) is the most common technique for the nondestructive assessment and analysis of the three-dimensional bone architecture. There were two papers using μCT to assess the effect of TBT on bone [16, 17]. However, they both used adult female rodents. Although osteopenia is more serious in women than men, there is a 20% osteoporotic fracture risk in white men [2]. Moreover, skeletal growth is rapid during adolescence, and exposure to EDs before and during puberty therefore results in greater deficits at a site than exposure after puberty [18]. In this study, we would furtherly assess the effect of TBT on bone based on femoral cancellous and cortical architecture using μCT, derived polar moment of inertia (Jo), ash content (mineral content), serum calcium ion (Ca^2+^) and inorganic phosphate (Pi) levels, and expression of genes related to adipogenesis and osteogenesis.

## Methods

### Reagents

TBT (purity ≥96%) was purchased from Sigma-Aldrich Chemicals Co. (St Louis, MO). Kits for determination of total protein, serum Ca^2+^ and Pi were from NanJing JianCheng Bioengineering Institute (Nanjing, China). The QIAamp RNA Blood Mini Kit and QuantiTect SYBR® Green RT-PCR Kits were from QIAGEN (regional headquarter, Shanghai for Asia). All other chemicals were of analytical grade and were obtained from commercial sources.

### Animals and treatment

21-day-old male SD rats were purchased from Beijing Vital River Laboratory Animal Technology Co., Ltd. [License No. SCXK (Jing) 20,160,011]. After 3 days of acclimatization, rats were randomly assigned into four groups (10 rats per group) based on body weight to achieve similar average weights in different groups. Rats were treated with corn oil or TBT (0.5, 5 or 50 μg·kg^− 1^) by gavage once every 3 days from ages of 24 d to 84 d, while the established no observable adverse effect level (NOAEL) of TBT was 25 μg·kg^− 1^·day^− 1^ based on immunological effects [19]. All rats were killed 1 day after the final gavage (85 d). All treatments of rats in this study were performed humanely in accordance with the National Institutes of Health’s Guide for the Care and Use of Laboratory Animals and followed the principles described in the “Use of Animals in Toxicology,” which were approved by the Ethics Committees of the School of Public Health, Shandong University, in March 2019. The identification code was 20,190,204.

### Preparation of serum and femurs

Urethane was dissolved in saline solution at a final concentration of 20% (w/v). The rats were anesthetized with urethane (~ 1.2 g·kg^− 1^) by introperitoneal injection. After reaching the surgical level of anesthesia, blood was obtained from the aorta ventralis. The serum was separated and stored at − 80 °C for further analysis. Both femurs were isolated modestly, and then the muscular tissue was removed. Five left femora were used to assess gene expression, and the other left femora were stored at − 20 °C for ash content analysis. All right femurs were weighed and measured as described in a previous study [15], and fixed in 4% (vol/vol) paraformaldehyde for further research.

### RNA isolation and quantitative real-time reverse transcription-polymerase chain reaction (QPCR)

Bone marrow (BM) was flushed from the left femur (*n* = 5 per group), and strained through a 70-μm cell strainer. After centrifugation, total RNA was extracted using the QIAamp RNA Blood Mini Kit (Qiagen, Valencia, CA) according to the manufacturer’s recommendation. The concentration and quality of RNA were determined with a NanoDrop 2000c spectrophotometer (Gene Company Limited, USA). After quality inspection, RNA was stored at − 80 °C for QPCR.

QPCR was performed using QuantiTect SYBR® Green RT-PCR Kits (QIAGEN) with reactions scaled to 25 μl, and 25 ng mRNA was used in each reaction. PCRs (in duplicate) were performed using a 7500 Fast Real-Time PCR System (Applied Biosystems, Carlsbad, CA) with a program consisting of 95 °C for 2 min, 40 cycles of 95 °C for 10 s and then 60 °C for 20 s and 72 °C for 30 s, followed by melting curve analysis. The primer sequences for each gene are listed in Table S1. Genes of interest were normalized to the GAPDH gene in the same sample following the 2^–ΔΔ^CT method [20]. Primers were designed using Primer-BLAST (NCBI) or Primer5 software (Rozen and Skaletsky 2000), and were verified by gradient amplification and melting curve analysis.

### Micro-computed tomography (μCT)

The fixed femurs were scanned with a Scanco μCT100 scanner (Scanco Medical AG, Bassersdorf, Switzerland), at a 20 μm isotropic resolution using an integration time of 300 ms, energy of 70 kVp, and intensity of 200 μA. Gaussian filtering (sigma = 0.8, support = 1) was used to reduce background noise. For analyses of trabecular bone within the distal femur, the specimen was scanned in 700 slices and a region of interest (ROI) of trabecular bone was selected between 230 slices and 300 slices to the growth plate. Trabecular bone parameters of the femoral metaphysis, including bone volume fraction (BV/TV), connectivity density (Conn.D), structural model index (SMI), trabecular number (Tb.N), trabecular thickness (Tb.Th), and trabecular separation (Tb.Sp), were determined using Scanco’s 3D analysis tools (direct model). For cortical bone analysis, the femur specimens were scanned similarly. One millimeter thick sections immediately distal to the mid-diaphysis were used as ROI. In addition, total cross sectional area (TCS.Ar), cortical cross sectional area (Ct.Ar), cortical thickness (Ct.th), periosteal circumference (Ps.Cf), endocortical circumference (Ec.Cf) and marrow area (Ma.Ar) were obtained from the analysis.

### Calculation of Jo

Jo was calculated as the medio-lateral (IML) + antero-posterior (IAP) axes, following the method of Jepsen et al. [21].

### Ash content

Five left femora per group were used to detect the ash content. The dried, and ash weights were determined as described previously [21]. Ash content was determined as the ash weight normalized for hydrated weight.

### Serum parameters

Serum Ca^2+^ and Pi levels were detected by colorimetry, following the manufacturer’s instructions.

### Statistical analysis

The statistical analyses were performed by SPSS software, version 21.0 for Windows (SPSS Inc., Chicago, IL, USA). Two-tailed Student’s t-test was used to analyze the bone structural phenotype and Jo, which were compared between the two groups. Other indicators were analyzed by ANOVA. The significance level was set at 0.05. Once significance was established *(P* < 0.05), Dunnett’s or Dunnett’s T3 test was performed to make multiple comparisons among the groups based on the homogeneity of variance.

## Results

### Effect of TBT on body weight and femur

**Fig. 1:**
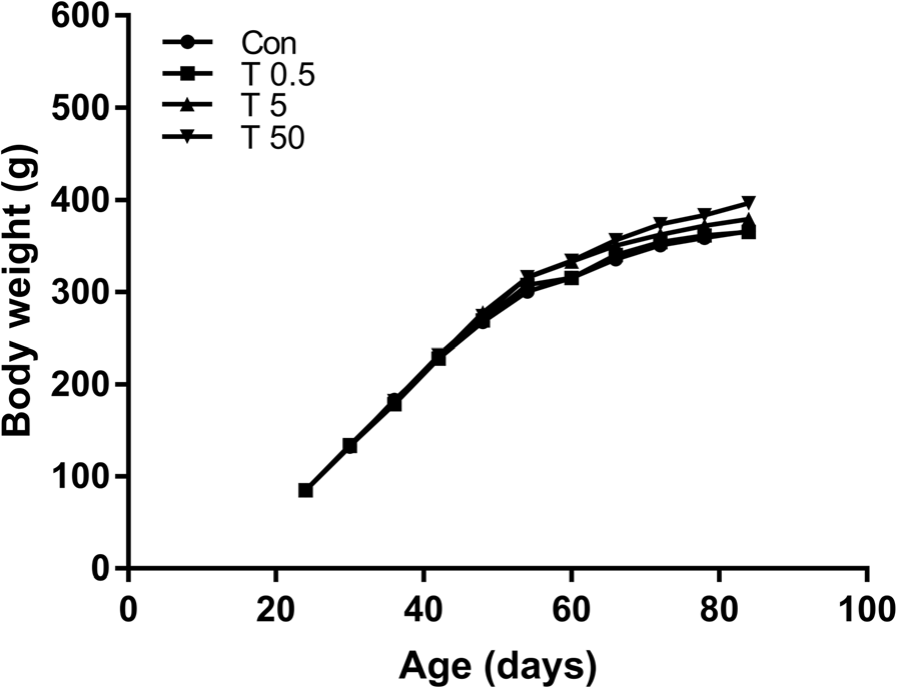
Effects of TBT exposure on body weight. The body weight of rats was not affected by TBT exposure at all time points, *n* = 10

The mean body-weight of rats is shown in Fig.1. Rat body weights increased with prolonged feeding time, however, exposure to TBT caused no significant effects on the body weights at all time points observed compared to the control. Consistent with the body weight, TBT-treatment caused no significant differences in femoral length and weight at the end of the experiment (Table 1).

**Table 1:** Bone length and bone weight of rats

Group (μg·kg^− 1^)	Bone length (mm)	Bone weight (g)
Control	36.47 ± 1.35	0.84 ± 0.10
TBTCl 0.5	36.15 ± 1.36	0.85 ± 0.08
TBTCl 5	36.06 ± 0.95	0.86 ± 0.08
TBTCl 50	36.00 ± 0.99	0.85 ± 0.06

Date was shown as means ± SD, *n* = 10

### Effect of TBT on the mRNA expression of genes involved in adipogenesis and osteogenesis in BM

PPARγ is a key transcriptional regulator of fat formation [22], while Fabp4 and Angptl4 are their target genes [23, 24]. Runx2 and ALP are early osteogenic markers [25, 26] and osteocalcin (OC) is a late osteoblast differentiation marker [27].

**Fig. 2:**
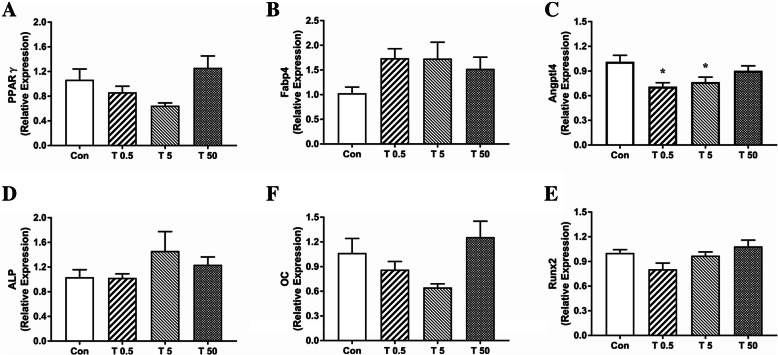
Effect of TBT on genes expression related to adipogenesis and osteogenesis. Expression of PPARγ (**a**), Fabp4 (**b**), Angptl4 (**c**), ALP (**d**), OC (**e**) and Runx2 (**f**) under different treatments. Data were presented as mean ± SEM, *n* = 5, *P<0.05, compared with control

Analysis with QPCR analysis showed no notable TBT-related changes in the expression of PPARγ, Fabp4, ALP and OC at 85 d (Fig.2). The relative expression of Fabp4 showed 1.69-, 1.68- and 1.48-fold increases in respective TBT groups, but the change was not significant (*P* = 0.153). Although the expression of Angptl4 showed a significant decrease in the 0.5 μg·kg^− 1^ and 5 μg·kg^− 1^ TBT groups compared with the control (*P* < 0.05), the degree of the decline was minor (− 29.79% and − 24.25%, respectively).

### Effect of TBT on microstructure of femur

**Fig. 3:**
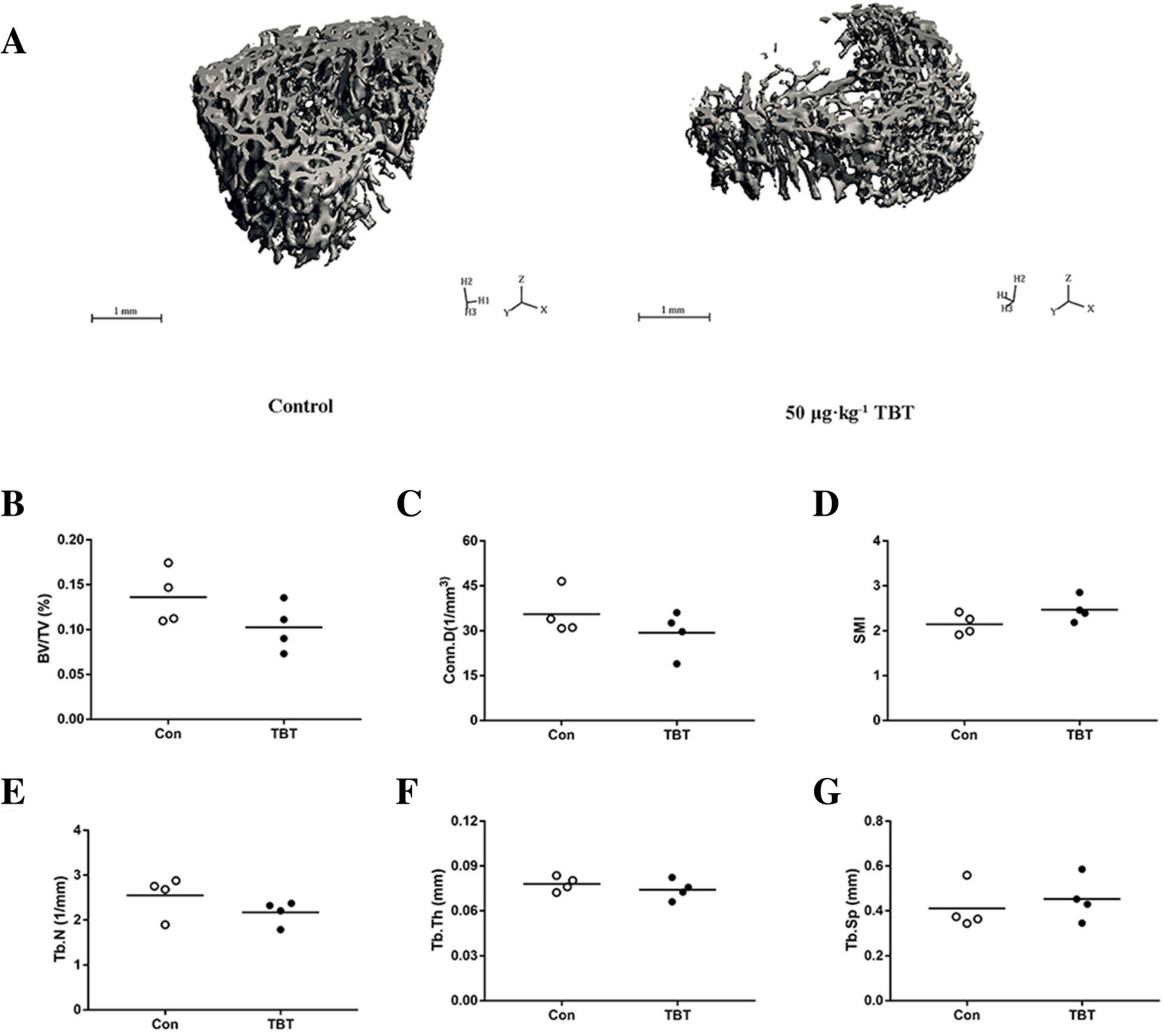
Effects of TBT on femoral metaphysis of rat. Representative μCT images of trabecular architecture in the distal metaphysis form control and the 50 μg·kg^− 1^ TBT rats (**a**), BV/TV (**b**), Conn. D (**c**), SMI (**d**), Tb. N (**e**), Tb. Th (**f**), Tb. Sp (**g**). Data were presented from individual rat, and the mean is indicated by a line; *n* = 4

**Fig. 4:**
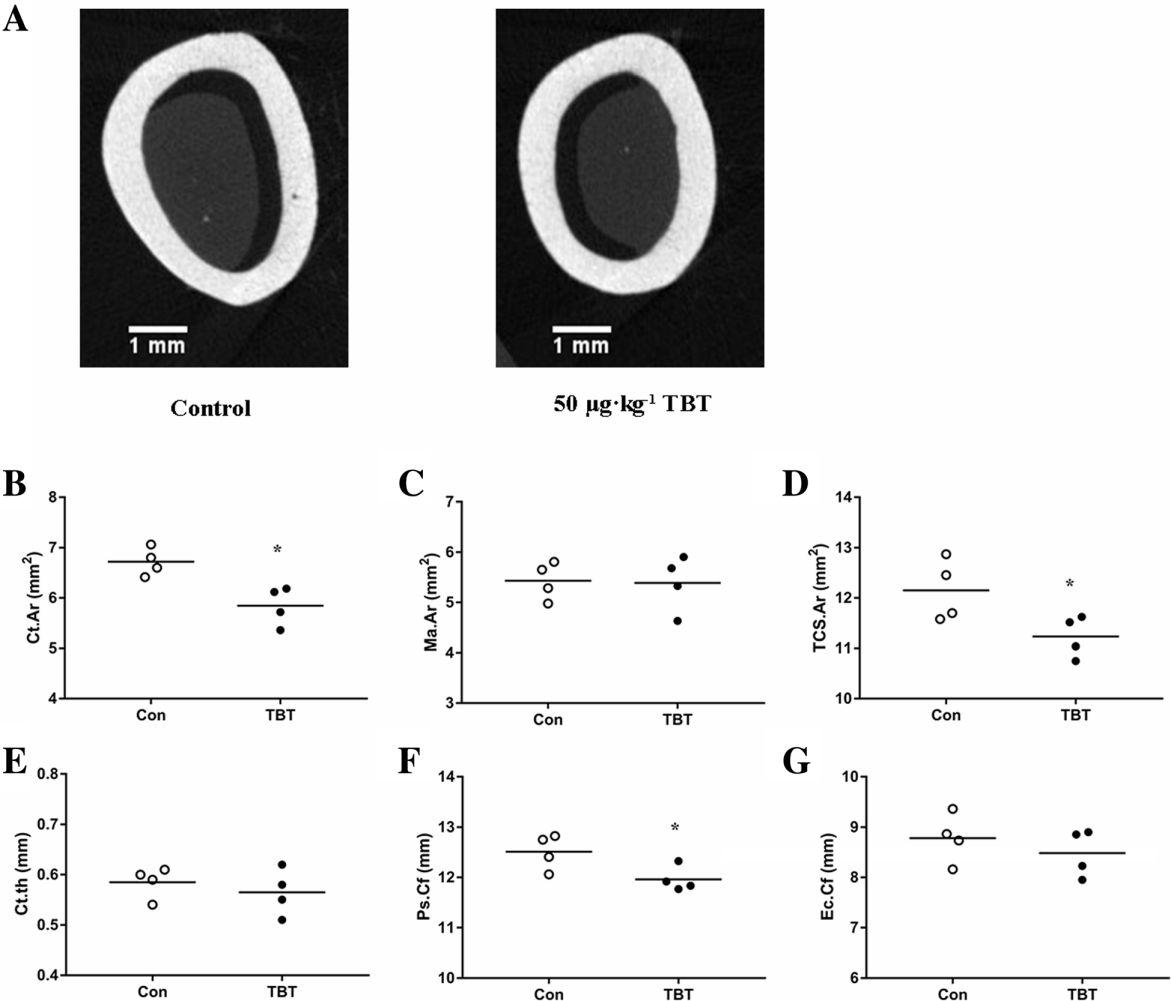
Effect of TBT on femoral diaphysis of rat. Representative μCT images of femoral diaphysis from control and the 50 μg·kg^− 1^ TBT group (**a**), Ct. Ar (**b**), Ma. Ar (**c**), TCS. Ar (**d**), Ct.th (**e**), Ps. Cf (**f**), Ec. Ct (**g**). Data were presented from individual rat, and the mean is indicated by a line; *n* = 4, *P < 0.05, compared with control

Quantitative measures of trabecular bone quality showed a decreasing trend in the BV/TV (− 24.60%, *P* = 0.153) along with a decreasing trend in Conn. D (− 17.56%, *P* = 0.277), Tb. N (− 14.92%, *P* = 0.193), and a subtle increase in Tb. Sp (+ 10.53%, *P* = 0.390) and SMI (+ 15.17%, *P* = 0.124), in TBT-treated rats compared to controls, although the differences were not significant (Fig.3). In contrast, the analysis of the mid-diaphysis showed that TBT-treated rats had a smaller Ct. Ar, Th. Ar and Ps. Cf than those of their control counterparts (Fig.4; *P* < 0.05), but had no effects on other indexes.

### Effect of TBT on Jo

**Fig. 5:**
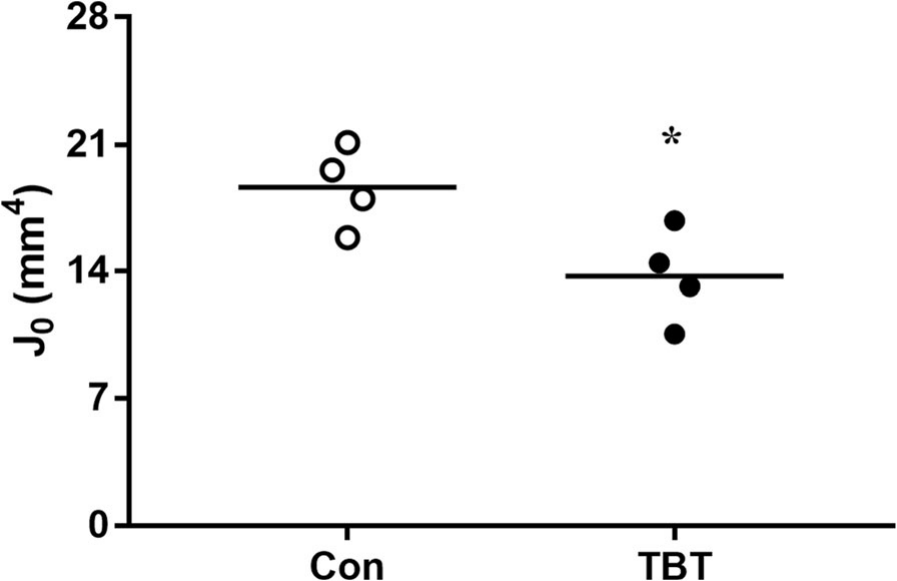
Effect of TBT on polar moment of inertia (Jo). Data are presented from individual rat, and the mean is indicated by a line; *n* = 4, *P < 0.05, compared with control

Jo was significantly reduced in 50 μg·kg^− 1^ TBT-treated rats, compared with control rats (*P* < 0.05, Fig.5).

### Effect of TBT on ash content

**Fig. 6:**
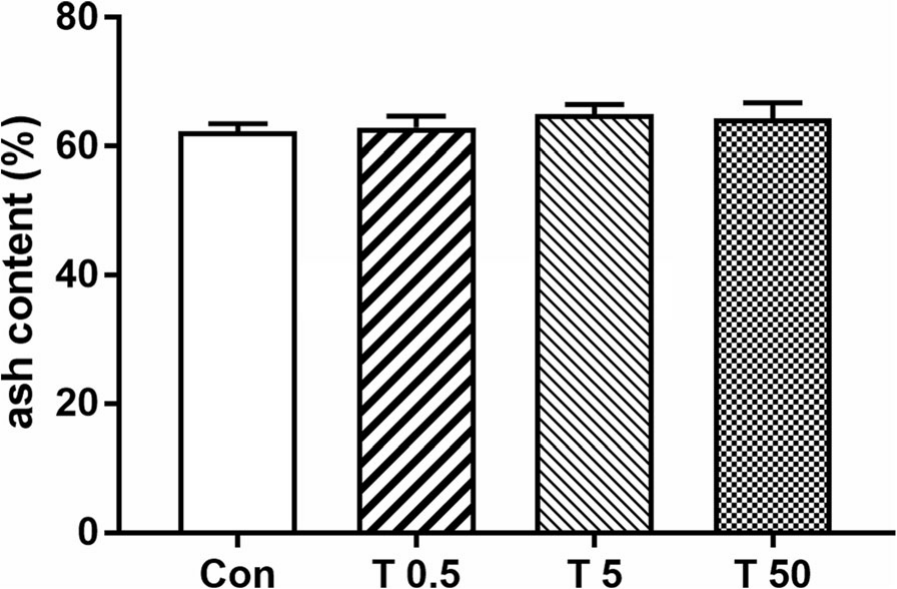
Effect of TBT on femoral ash content of rat. Data were presented as mean ± SEM, *n* = 5

There was no significant difference in ash content between the TBT groups and the control group (Fig.6).

### Effect of TBT on serum Ca^2+^ and pi levels

**Fig. 7:**
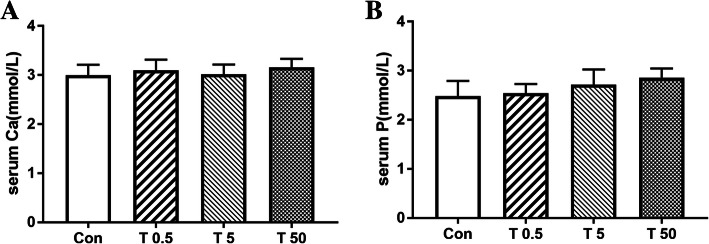
Effects of TBT on serum calcium and phosphorus of rats. Expression of serum calcium (**a**) and phosphorus (**b**) under different treatments. Data were presented as mean ± SEM, *n* = 10

As shown in Fig.7, there were no significant changes in serum levels of Ca^2+^ and Pi between the control and TBT treated rats.

## Discussion

Consistent with our previous result on the BMD of the femur, μCT analysis revealed that the effect of TBT was more significant on the diaphysis than on the cancellous diaphysis in the femur of rats in this study. However, the study of Watt et al. observed a reduced femoral cortical cross-sectional area and thinner cortex with increased cancellous Tb. Th, Tb. N, and BV/TV in TBT-treated mice [16]. The discrepancies between the two studies might be explained by (1) the different dose. EDs could exhibit complex dose-response curves, and they might produce different effects at extremely low concentrations [28–30]. The highest concentration used in our study was 200-fold lower than the dose used in the study by Watt et al.; (2) The different species and sexes. Several systematic studies have revealed that femoral morphology and composition differ in genetic variation, sex and age [31–33]; (3) The different exposure times. Our exposure time included puberty of rats while that of Watt et al. only included the adult stage. Notably, skeletal growth is rapid during adolescence [34], in which endochondral and intramembranous ossification are produced simultaneously, while the process of endochondral ossification disappears after puberty [35]. Hence, the effect of TBT in this study includes the effect on skeletal development, while such an effect in the study of Watt et al. [16] is only on bone remodeling. As a long bone, the femoral diaphysis is a hollow cylinder whose size and shape can be influenced by the relative amounts of bone deposition and resorption on the periosteal and endosteal surfaces [36–39]. Therefore, a significantly smaller periosteal expansion and marrow infilling might lead to a smaller Ct. Ar, Th. Ar and Ps. Cf, which reflected the relative contribution of osteoblasts and osteoclasts working on them [33]. Indeed, in vitro studies showed that TBT not only inhibited osteoblasts [40, 41] but also suppressed osteoclastogenesis and resorptive activity of osteoclasts [16, 42].

Mesenchymal stem cells (MSCs) are multipotent cells contributing to osteoblast and adipocyte progenies in adult bone marrow [43]. As common progenitor cells of adipocytes and osteoblasts, the commitment towards these lineages is classically considered to be inversely related [44]. As a dual RXRα/β and PPARγ agonist [22], TBT could reprogram BM-MSCs towards adipogenesis at the cost of osteogenesis in vitro [40, 41] and in vivo [45]. In contrast, TBT did not obviously change the expression of PPARγ and its target genes in this study. Consistent with the response of genes involved in adipogenesis, genes associated osteogenesis (ALP, OC and Runx2) were not significantly changed in this study. Since the relative expressive fold of Angptl4 expression was marginal only in the 0.5 and 5 μg·kg^− 1^ TBT groups, and had no effects in the 50 μg·kg^− 1^ TBT group. These data revealed that the impaired effects of TBT on the mesenchymal differentiation disappeared at 85d, suggesting that TBT treatment might have no persistent effects on the adipo-osteogenic balance in the BM of rats at the transcriptional level. This no residual effects might be partly due to the resistance produced by rats to TBT. One recent paper showed that feeding young mice a high-fat diet (HFD) significantly increased the CD45^−^CD31^−^Sca1^+^CD24^+^ (a tri-potent population with stem cell-like characteristics) and adipogenic progenitor cells (APCs) frequencies for 1 day but not for 10 days [46]. Our recent study showed that TBT treatment resulted in a dose-dependent increase in lipid accumulation and adipocyte number in the BM of the femur [15]. In addition, our previous study demonstrated that TBT-induced functional perturbations of the gut microbiome were similar to the HFD styles [47]. Therefore, whether the effect of TBT on APC proliferation is similar to that of HFD needs to be further investigated.

The different effects of TBT between this study and in vitro studies [40, 41] were at least partially were explained by the compensation effect of rats, because experimental animals can exert feedback mechanisms to counteract harmful effects. However, one paper [45] observed up-regulated MSCs expression of genes involved in lipogenesis in all three subsequent generations after ancestral perinatal TBT exposure. Preimplantation development is a major developmental period of epigenetic reprogramming of the genome in mammalians [48]. Indeed, exposure to TBT throughout pregnancy and lactation induced genome-wide alterations in methylation and altered the expression of metabolism-relevant genes in unexposed male descendants [49]. However, genomic methylation patterns are generally stable and heritable in somatic differentiated cells [48]. Therefore, the different exposure times might be a plausible explanation for the discrepancy between that of Chamorro-García et al. [45] and ours.

Jo is a quantity used to predict an object’s ability to resist torsion. Because structures possessing the same Ct. Ar but different moments of inertia (e.g., a solid cylinder and a tube) will exhibit different mechanical characteristics in bending and torsion [50], so measuring Jo is necessary. Moreover, the level of Jo is consistent with bone strength [51]. Ct. Ar, Jo, and ash content together might explain 66–88% of the genetic variability in adult whole bone mechanical properties [21, 33]. In this study, treatment with 50 μg·kg^− 1^ TBT caused a significant decrease in Ct. Ar and Jo, but not on ash content, which might be one possible explanation for the reduced but no significantly different biomechanical strength change induced by TBT in our previous study [15].

The skeleton is made of collagen fibers that form a scaffold where mineralization is initiated by the accumulation of Ca^2+^ and Pi, mainly in the form of crystalline hydroxyapatite (HA) [52]. Calcium and phosphorus play critical roles in diverse biological processes including bone mineralization [53, 54]. TBT treatment resulted in no significant changes in the serum Ca^2+^ and Pi levels in this study, which was in accordance with the data of ash content.

## Conclusions

In conclusion, treatment with 50 μg·kg^− 1^ TBT caused a significant decrease in femoral Ct. Ar, Th. Ar and Jo of rat. All the data suggest that exposure to a low dose of TBT from the prepubertal to adult stage produces adverse effects on skeletal architecture and strength.

## Supplementary Information

**Additional file 1: Table S1.** Primers used for QPCR analysis of gene expression.

**Additional file 2.**

**Additional file 3.**

**Additional file 4.**

**Additional file 5.**

**Additional file 6.**

## Data Availability

All data generated and analyzed during this study are included in its supplementary information files.

## Abbreviations

APCs: Adipogenic progenitor cells
BM: Bone marrow
BMD: Bone mineral density
BV/TV: Bone volume fraction
Ca^2+^: Calcium ion
Conn.D: Connectivity density
Ct.Ar: Cortical cross sectional area
Ct.th: Cortical thickness
Ec.Cf: Endocortical circumference
EDs: Endocrine disruptor
HA: Hydroxyapatite
IAP: Antero-posterior axes
IML: Medio-lateral axes
Ma.Ar: Marrow area
MSCs: Mesenchymal stem cells
OC: Osteocalcin
Ps.Cf: Periosteal circumference
Pi: Phosphate
PPARγ: Peroxisome proliferator activated receptor γ
SMI: Structural model index
Tb.N: Trabecular number
Tb.Th: Trabecular thickness
TBT: Tributyltin chloride
Tb.Sp: Trabecular separation
TCS.Ar: Total cross sectional area
μCT: Micro-computed tomography

## References

[1] Rachner TD, Khosla S, Hofbauer LC (2011). Osteoporosis: now and the future. Lancet.

[2] Porter JL, Varacallo M: Osteoporosis. [Updated 2020 Nov 21] In: StatPearls [Internet] Treasure Island (FL): StatPearls Publishing; 2020 Jan- Available from: https://www.ncbinlmnihgov/books/NBK441901/2020.

[3] Berry SD, Kiel DP, Colón-Emeric C (2019). Hip fractures in older adults in 2019. Jama.

[4] Seeman E (2002). Pathogenesis of bone fragility in women and men. Lancet.

[5] Prada D, López G, Solleiro-Villavicencio H, Garcia-Cuellar C, Baccarelli AA (2020). Molecular and cellular mechanisms linking air pollution and bone damage. Environ Res.

[6] Toni R, Di Conza G, Barbaro F, Zini N, Consolini E, Dallatana D, Antoniel M, Quarantini E, Quarantini M, Maioli S (2020). Microtopography of immune cells in osteoporosis and bone lesions by endocrine disruptors. Front Immunol.

[7] van Zwol-Janssens C, Trasande L, Asimakopoulos AG, Martinez-Moral MP, Kannan K, Philips EM, Rivadeneira F, Jaddoe VWV, Santos S (2020). Fetal exposure to bisphenols and phthalates and childhood bone mass: a population-based prospective cohort study. Environ Res.

[8] Yang Z, Shi J, Guo Z, Chen M, Wang C, He C, Zuo Z (2019). A pilot study on polycystic ovarian syndrome caused by neonatal exposure to tributyltin and bisphenol a in rats. Chemosphere.

[9] Antizar-Ladislao B (2008). Environmental levels, toxicity and human exposure to tributyltin (TBT)-contaminated marine environment. Environ Int.

[10] Mattos Y, Stotz WB, Romero MS, Bravo M, Fillmann G, Castro IB (2017). Butyltin contamination in northern Chilean coast: is there a potential risk for consumers?. Sci Total Environ.

[11] Takahashi S, Mukai H, Tanabe S, Sakayama K, Miyazaki T, Masuno H (1999). Butyltin residues in livers of humans and wild terrestrial mammals and in plastic products. Environ Pollut.

[12] Kannan K, Senthilkumar K, Giesy JP (1999). Occurence of butyltin compounds in human blood. Environ Sci Technol.

[13] Adeeko A, Li D, Forsyth DS, Casey V, Cooke GM, Barthelemy J, Cyr DG, Trasler JM, Robaire B, Hales BF (2003). Effects of in utero tributyltin chloride exposure in the rat on pregnancy outcome. Toxicol Sci.

[14] Tsukamoto Y, Ishihara Y, Miyagawa-Tomita S, Hagiwara H (2004). Inhibition of ossification in vivo and differentiation of osteoblasts in vitro by tributyltin. Biochem Pharmacol.

[15] Yao W, Wei X, Guo H, Cheng D, Li H, Sun L, Wang S, Guo D, Yang Y, Si J (2020). Tributyltin reduces bone mineral density by reprograming bone marrow mesenchymal stem cells in rat. Environ Toxicol Pharmacol.

[16] Watt J, Baker AH, Meeks B, Pajevic PD, Morgan EF, Gerstenfeld LC, Schlezinger JJ (2018). Tributyltin induces distinct effects on cortical and trabecular bone in female C57Bl/6J mice. J Cell Physiol.

[17] Resgala LCR, Santana HS, Portela BSM, Zanovello MVS, da Costa CS, Niño OMS, Endlich Bicudo N, Dos Santos DZ, Lang Podratz P, da Cunha M, Oliveira ALA, Ceotto Freitas Lima L, Gomes-Rochette NF, Rangel LBA, Graceli JB, Silva IV (2019). Effects of Tributyltin (TBT) on rat bone and mineral metabolism. Cell Physiol Biochem.

[18] Bass S, Delmas PD, Pearce G, Hendrich E, Tabensky A, Seeman E (1999). The differing tempo of growth in bone size, mass, and density in girls is region-specific. J Clin Invest.

[19] Vos JG, De Klerk A, Krajnc EI, Van Loveren H, Rozing J (1990). Immunotoxicity of bis (tri-n-butyltin) oxide in the rat: effects on thymus-dependent immunity and on nonspecific resistance following long-term exposure in young versus aged rats. Toxicol Appl Pharmacol.

[20] Livak KJ, Schmittgen TD (2001). Analysis of relative gene expression data using real-time quantitative PCR and the 2(−Delta Delta C(T)) method. Methods.

[21] Jepsen KJ, Akkus OJ, Majeska RJ, Nadeau JH (2003). Hierarchical relationship between bone traits and mechanical properties in inbred mice. Mamm Genome.

[22] Kanayama T, Kobayashi N, Mamiya S, Nakanishi T, Nishikawa J (2005). Organotin compounds promote adipocyte differentiation as agonists of the peroxisome proliferator-activated receptor gamma/retinoid X receptor pathway. Mol Pharmacol.

[23] Burris TP, Pelton PD, Zhou L, Osborne MC, Cryan E, Demarest KT (1999). A novel method for analysis of nuclear receptor function at natural promoters: peroxisome proliferator-activated receptor gamma agonist actions on aP2 gene expression detected using branched DNA messenger RNA quantitation. Mol Endocrinol.

[24] Yoon JC, Chickering TW, Rosen ED, Dussault B, Qin Y, Soukas A, Friedman JM, Holmes WE, Spiegelman BM (2000). Peroxisome proliferator-activated receptor gamma target gene encoding a novel angiopoietin-related protein associated with adipose differentiation. Mol Cell Biol.

[25] Beier EE, Maher JR, Sheu TJ, Cory-Slechta DA, Berger AJ, Zuscik MJ, Puzas JE (2013). Heavy metal lead exposure, osteoporotic-like phenotype in an animal model, and depression of Wnt signaling. Environ Health Perspect.

[26] Kawane T, Qin X, Jiang Q, Miyazaki T, Komori H, Yoshida CA, Matsuura-Kawata V, Sakane C, Matsuo Y, Nagai K, Maeno T, Date Y, Nishimura R, Komori T (2018). Runx2 is required for the proliferation of osteoblast progenitors and induces proliferation by regulating Fgfr2 and Fgfr3. Sci Rep.

[27] Owen TA, Aronow M, Shalhoub V, Barone LM, Wilming L, Tassinari MS, Kennedy MB, Pockwinse S, Lian JB, Stein GS (1990). Progressive development of the rat osteoblast phenotype in vitro: reciprocal relationships in expression of genes associated with osteoblast proliferation and differentiation during formation of the bone extracellular matrix. J Cell Physiol.

[28] Kaiser J (2003). Hormesis. Sipping from a poisoned chalice. Science.

[29] Vandenberg LN, Colborn T, Hayes TB, Heindel JJ, Jacobs DR, Lee DH, Shioda T, Soto AM, vom Saal FS, Welshons WV (2012). Hormones and endocrine-disrupting chemicals: low-dose effects and nonmonotonic dose responses. Endocr Rev.

[30] Gore AC, Chappell VA, Fenton SE, Flaws JA, Nadal A, Prins GS, Toppari J, Zoeller RT (2015). EDC-2: the Endocrine Society's second scientific statement on endocrine-disrupting chemicals. Endocr Rev.

[31] Glatt V, Canalis E, Stadmeyer L, Bouxsein ML (2007). Age-related changes in trabecular architecture differ in female and male C57BL/6J mice. J Bone Miner Res.

[32] Halloran BP, Ferguson VL, Simske SJ, Burghardt A, Venton LL, Majumdar S (2002). Changes in bone structure and mass with advancing age in the male C57BL/6J mouse. J Bone Miner Res.

[33] Price C, Herman BC, Lufkin T, Goldman HM, Jepsen KJ (2005). Genetic variation in bone growth patterns defines adult mouse bone fragility. J Bone Miner Res.

[34] Braun JM (2017). Early-life exposure to EDCs: role in childhood obesity and neurodevelopment. Nat Rev Endocrinol.

[35] Ballabriga A (2000). Morphological and physiological changes during growth: an update. Eur J Clin Nutr.

[36] van der Meulen MC, Beaupré GS, Carter DR (1993). Mechanobiologic influences in long bone cross-sectional growth. Bone.

[37] Seeman E (1997). From density to structure: growing up and growing old on the surfaces of bone. J Bone Miner Res.

[38] Duan Y, Beck TJ, Wang XF, Seeman E (2003). Structural and biomechanical basis of sexual dimorphism in femoral neck fragility has its origins in growth and aging. J Bone Miner Res.

[39] Sontag W (1986). Quantitative measurement of periosteal and cortical-endosteal bone formation and resorption in the midshaft of female rat femur. Bone.

[40] Yanik SC, Baker AH, Mann KK, Schlezinger JJ (2011). Organotins are potent activators of PPARgamma and adipocyte differentiation in bone marrow multipotent mesenchymal stromal cells. Toxicol Sci.

[41] Baker AH, Watt J, Huang CK, Gerstenfeld LC, Schlezinger JJ (2015). Tributyltin engages multiple nuclear receptor pathways and suppresses osteogenesis in bone marrow multipotent stromal cells. Chem Res Toxicol.

[42] Yonezawa T, Hasegawa S, Ahn JY, Cha BY, Teruya T, Hagiwara H, Nagai K, Woo JT (2007). Tributyltin and triphenyltin inhibit osteoclast differentiation through a retinoic acid receptor-dependent signaling pathway. Biochem Biophys Res Commun.

[43] Chen Q, Shou P, Zheng C, Jiang M, Cao G, Yang Q, Cao J, Xie N, Velletri T, Zhang X, Xu C, Zhang L, Yang H, Hou J, Wang Y, Shi Y (2016). Fate decision of mesenchymal stem cells: adipocytes or osteoblasts?. Cell Death Differ.

[44] Kawai M, Rosen CJ (2010). PPARγ: a circadian transcription factor in adipogenesis and osteogenesis. Nat Rev Endocrinol.

[45] Chamorro-Garcia R, Sahu M, Abbey RJ, Laude J, Pham N, Blumberg B (2013). Transgenerational inheritance of increased fat depot size, stem cell reprogramming, and hepatic steatosis elicited by prenatal exposure to the obesogen tributyltin in mice. Environ Health Perspect.

[46] Ambrosi TH, Scialdone A, Graja A, Gohlke S, Jank AM, Bocian C, Woelk L, Fan H, Logan DW, Schurmann A (2017). Adipocyte accumulation in the bone marrow during obesity and aging impairs stem cell-based hematopoietic and bone regeneration. Cell Stem Cell.

[47] Guo H, Yan H, Cheng D, Wei X, Kou R, Si J (2018). Tributyltin exposure induces gut microbiome dysbiosis with increased body weight gain and dyslipidemia in mice. Environ Toxicol Pharmacol.

[48] Reik W, Dean W, Walter J (2001). Epigenetic reprogramming in mammalian development. Science.

[49] Chamorro-Garcia R, Diaz-Castillo C, Shoucri BM, Käch H, Leavitt R, Shioda T, Blumberg B (2017). Ancestral perinatal obesogen exposure results in a transgenerational thrifty phenotype in mice. Nat Commun.

[50] van der Meulen MC, Jepsen KJ, Mikić B (2001). Understanding bone strength: size isn't everything. Bone.

[51] Wergedal JE, Veskovic K, Hellan M, Nyght C, Balemans W, Libanati C, Vanhoenacker FM, Tan J, Baylink DJ, Van Hul W (2003). Patients with Van Buchem disease, an osteosclerotic genetic disease, have elevated bone formation markers, higher bone density, and greater derived polar moment of inertia than normal. J Clin Endocrinol Metab.

[52] Anderson HC (1989). Mechanism of mineral formation in bone. Lab Investig.

[53] Peacock M (2010). Calcium metabolism in health and disease. Clin J Am Soc Nephrol.

[54] Berndt T, Kumar R (2009). Novel mechanisms in the regulation of phosphorus homeostasis. Physiology (Bethesda).

