# Supplementary material for: Tributyltin perturbs femoral cortical architecture and polar moment of inertia in rat
Source: BMC Musculoskelet Disord. 2021 May 7;22:427. doi: 10.1186/s12891-021-04298-2 (PMC8106170; doi:10.1186/s12891-021-04298-2)
Supplement: Supplementary file 1 — Additional file 1: Table S1. Primers used for QPCR analysis of gene expression. [file 12891_2021_4298_MOESM1_ESM.docx]

**Table S1. Primers used for QPCR analysis of gene expression**

| **Gene** | **Primer seq (5'-3')** | | **Amplicon size (bp)** | **GenBank** |
| --- | --- | --- | --- | --- |
| PPARγ | F | GCTGTTATGGGTGAAACTCTGG | 74 | NM_013124.3 |
|  | R | ATAGGCAGTGCATCAGCGAA |  |  |
| Fabp4 | F | AGAAGGGGACTTGGTCGTCA | 76 | NM_053365.1 |
|  | R | TTCCACGCCCAGTTTGAAGG |  |  |
| Angptl4 | F | ACCTGCAGCCATTCCAATCT | 104 | NM_199115.2 |
|  | R | TAGCGGCCCTTCCATGTTTT |  |  |
| Runx2 | F | TGGTGTTGACGCTGATGGAA | 88 | NM_001278484.2 |
|  | R | ATAGGGTCGCCAGACAGACT |  |  |
| ALP | F | CACGGCGTCCATGAGCAGAAC | 83 | J03572.1 |
|  | R | CAGGCACAGTGGTCAAGGTTGG |  |  |
| OC | F | CCGTTTAGGGCATGTGTTGC | 98 | M11777.1 |
|  | R | TTTCGAGGCAGAGAGAGGGA |  |  |
| GAPDH | F | AGTGCCAGCCTCGTCTCATA | 133 | NM_017008.4 |
|  | R | ATGAAGGGGTCGTTGATGGC |  |  |

Notice: F, forward; R, reverse.
